# *Enterobacter asburiae* Strain L1: Complete Genome and Whole Genome Optical Mapping Analysis of a Quorum Sensing Bacterium

**DOI:** 10.3390/s140813913

**Published:** 2014-07-30

**Authors:** Yin Yin Lau, Wai-Fong Yin, Kok-Gan Chan

**Affiliations:** Division of Genetics and Molecular Biology, Institute of Biological Sciences, Faculty of Science, University of Malaya, Kuala Lumpur 50603, Malaysia; E-Mails: yinyinlau88@yahoo.com (Y.Y.L.); yinwaifong@yahoo.com (W.-F.Y.)

**Keywords:** *Enterobacter asburiae*, quorum sensing, *N*-acyl homoserine lactone synthase, food spoilage, food safety, virulence factors, next-generation sequencing technology

## Abstract

*Enterobacter asburiae* L1 is a quorum sensing bacterium isolated from lettuce leaves. In this study, for the first time, the complete genome of *E. asburiae* L1 was sequenced using the single molecule real time sequencer (PacBio RSII) and the whole genome sequence was verified by using optical genome mapping (OpGen) technology. In our previous study, *E. asburiae* L1 has been reported to produce AHLs, suggesting the possibility of virulence factor regulation which is quorum sensing dependent. This evoked our interest to study the genome of this bacterium and here we present the complete genome of *E. asburiae* L1, which carries the virulence factor gene *virK*, the *N*-acyl homoserine lactone-based QS transcriptional regulator gene *luxR* and the *N*-acyl homoserine lactone synthase gene which we firstly named *easI*. The availability of the whole genome sequence of *E. asburiae* L1 will pave the way for the study of the QS-mediated gene expression in this bacterium. Hence, the importance and functions of these signaling molecules can be further studied in the hope of elucidating the mechanisms of QS-regulation in *E. asburiae*. To the best of our knowledge, this is the first documentation of both a complete genome sequence and the establishment of the molecular basis of QS properties of *E. asburiae*.

## 1. Introduction

*Enterobacter asburiae* is a Gram-negative, facultative anaerobic, oxidase negative, non-motile and non-pigmented rod-shaped species of the *Enterobacteriaceae* family that has been isolated from soil, water and food products [1–3]. It is also known as the epiphytic bacterium [4], which either has a parasitism or commensalism relationship with the plant host [5]. It has been reported as a quorum sensing (QS) bacterium [3] that is able to communicate via secretion of signaling molecules called autoinducers. QS regulates the expression of certain genes in response to the bacterial population density, *i.e.*, when a threshold amount of the autoinducers is detected [6,7], cooperative activities involved in survival and successful colonization such as the exoenzyme secretion, symbiosis, biofilm formation, sporulation, virulence, antibiotic production, bioluminescence and conjugation are activated [8,9]. Signaling via *N*-acyl homoserine lactones (AHLs) is the paradigm for QS in *Proteobacteria* [8]. These molecules, which consist of 4- to 18-carbon side chain linked to a lactone ring [10] are synthesized by AHL synthase (LuxI homologs) using *S*-adenosylmethionine (SAM) and acylated acyl carrier protein (Acyl-ACP) as the substrates [11].

Studies have shown that *Enterobacteriaceae* are commonly associated with food spoilage as well as food poisoning [12,13]. It is believed that the availability of AHL-regulated systems in the microbes could be the causative factor responsible for the toxicity of food products, deterioration of taste and texture, and ultimately, food safety threat [14]. Lipolytic, proteolytic, pectinolytic, and chitinolytic activities are among the traits that are possibly regulated by QS [15]. Consequently, study on the potential role of QS in food safety and food spoilage has provided a very important insight into food microbiology in order to generate useful information to reduce or prevent spoilage reactions as well as control the expression of virulence factors [16]. In depth investigations on bacterial QS properties could potentially offer solutions to resolve the food safety issues whilst improving human health.

As seen in recent years, genome-wide scale computational analysis is widely been used as a backbone to foster novel discovery in biomedical research. This high demand of low-cost sequencing has driven the rapid development of high-throughput sequencing. Next generation sequencing (NGS) technology offers rapid insights at the genome level at a decreasing cost and hence will soon become a common platform for bacterial genome study [17]. Coupling with the commercialization of various affordable desktop sequencers and fast improved computing power, researchers are able to map bacteria genome within a short period of time. In this study, two different NGS technologies were applied to generate the whole genome sequence of *E. asburiae* L1, an AHL-producing strain isolated from lettuce leaves. The complete genome was annotated and the gene functions were predicted to search for genes of interest.

## 2. Experimental Section

### 2.1. Bacterial Source, Isolation and Culture

*E. asburiae* L1 isolated from lettuce leaves was identified and characterized by obtaining pure cultures on MacConkey agar (Scharlau, Scharlab, Barcelona, Spain). The pure culture was routinely maintained on LB (Luria Bertani, Merck, Whitehouse Station, NJ, USA) agar at 37 °C or incubated overnight at 37 °C agitated at 200 rpm in LB broth. *E. asburiae* was also maintained kept at −80 °C in 80% (v/v) glycerol.

### 2.2. Scanning Electron Microscopy Imaging

Scanning electron microscopy (SEM) observation of *E. asburiae* L1 was conducted on a TM3000 Analytical Tabletop Microscope (Hitachi, Brisbane, CA, USA). The bacterial pellets were fixed in 2.5% glutaraldehyde for at least 2 h before proceeding with two 0.1 M phosphate buffer washes. The fixed cells were then subjected to post fixation with 1% osmium tetroxide for at least an hour. After two post-fixation washes, a graded series of ethanol dehydration steps (50%, 75%, 95%, 100%, 100% ethanol, 10 min each) was performed before immersed the cells in Hexamethyldisilazane (HMDS) (Ted Pella, Redding, CA, USA) for another 10 min. The SEM preparation was completed by decanting the HMDS from the tube and letting the cells air-dry in a desiccator at room temperature. Prior to examination, the dried cells were mounted onto a SEM specimen stub with a double-sided sticky tape and subjected to gold coating.

### 2.3. Genomic DNA Extraction

The genomic DNA of *E. asburiae* L1 was extracted using Masterpure™ DNA purification kit (Epicenter, Illumina Inc., Madison, WI, USA) per the manufacturer's instructions. The quality of the extracted DNA was performed with Nanodrop Spectrophotometer (Thermo Scientific, Pittsburgh, PA, USA) and agarose gel electrophoresis while DNA quantification was carried out with a Qubit^®^ 2.0 Fluorometer (dsDNA High Sensitivity Assay Kit, Invitrogen, Carlsbad, CA, USA).

### 2.4. Library Preparation and Sequencing

DNA sequencing template was obtained from sheared genomic DNA using the Pacific Bioscience 10 kb SMRTbell library template preparation kit per the manufacturer's instructions (Pacific Biosciences, Menlo Park, CA, USA). The quality sizing analysis of DNA library was validated by Bioanalyzer 2100 high sensitivity DNA kit (Agilent Technologies, Inc., Santa Clara, CA, USA) prior to sequencing. PacBio RS II sequencing technology (Pacific Biosciences) was used as the sequencing platform. P4 chemistry was utilized, and the prepared library was sequenced on four single-molecule real-time (SMRT) cells.

### 2.5. Whole Genome Optical Mapping

A whole genome map of *E. asburiae* L1 was generated from the single DNA molecule with the automated Argus system (OpGen Inc., Gaithersburg, MD, USA). DNA extraction was performed based on the manufacturer's instructions. Purified DNA was then diluted to the appropriate concentration by performing the quality check using QCard (OpGen Inc.). The DNA molecules were filled through all the channels of the channel-forming device (CFD) on MapCards II (OpGen Inc.) through capillary action. The four reagent reservoirs were pipetted into their individual load ports according to the labeled with the corresponding reagent on the left side of the MapCard II. Digestion was performed with *Afl*II for 30 min while all the four reagents were dispensed and aspirated from the reaction chamber at appropriate times, volumes, and flow rates in the MapCard Processor. Upon completion, the MapCard II was placed in whole genome mapper to perform whole genome optical mapping.

### 2.6. Gene Prediction and Annotation

Genes were predicted using Prodigal 2.60 while gene annotation was performed using RAST [18] followed by visualization of the bacterial circular genome using DNAPlotter version 1.4 (Artemis 12.0, Sanger Institute, Hinxton, Cambridge, UK) and Gepard version 1.3 (Institute of Computational Biology, Neuherberg, Germany) [19]. Phylogenetic analysis was performed using MEGA version 5.2 [20].

## 3. Results and Discussion

### 3.1. Isolation and Characterization of E. asburiae L1

**Figure 1.**
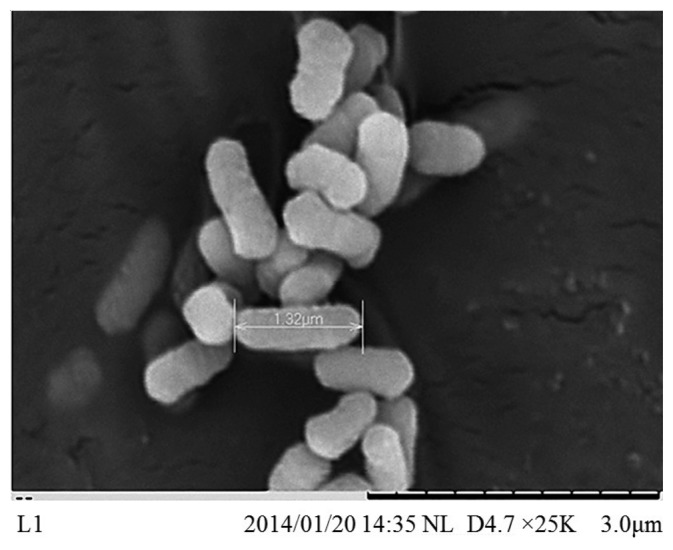
Scanning electron microscope image of *E. asburiae* L1. The size of the strain is approximately 1.32 μm (bar).

*E. asburiae* L1 was identified at the species level with score values above 2.3 using MALDI-TOF-MS (Bruker, Leipzig, Germany). Like other species classified in the *Enterobacteriaceae* family, *E. asburiae* L1 is a rod-shaped bacterium with approximately 1.32 μm in size (Figure 1). *E. asburiae* L1 lives in the mesophilic environment with its optimal temperature at 37 °C. In our previous study, *E. asburiae* L1 has been reported to produce AHLs [3], suggesting the possibility of virulence factors regulation by a QS mechanism.

### 3.2. Whole Genome Sequencing of E. asburiae L1

**Figure 2.**
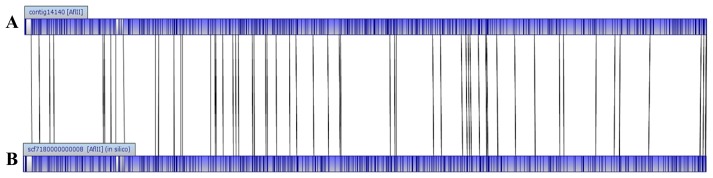
Alignment of (**A**) OpGen Sequence with (**B**) PacBio Sequence for *E. asburiae* L1.

**Figure 3.**
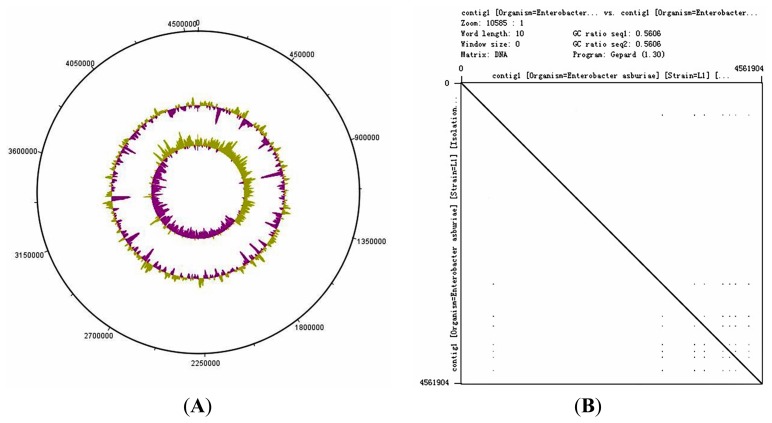
Circular representation of *E. asburiae* L1. The figure was constructed by (**A**) DNAPlotter version 1.4 and (**B**) Gepard version 1.3. The GC skew was shown in the most inner layer while the GC plot was shown in the second lane counting from the outer most lane. The genome size of *E. asburiae* L1 was 4.5 Mbp. The straight line of dotplot generated from Gepard further supported the circular representation of this genome.

The genome size of *E. asburiae* L1 is 4.5 Mbp. The PacBio sequencing platform generated an output data with average genome coverage of 216.24×. *De novo* assembly of the insert reads was performed with the Hierarchical Genome Assembly Process (HGAP) algorithm in SMRT Portal (version 2.1.1), in which the genome sequence of *E. asburiae* strain L1 was assembled into a GC-rich (56.1%) single contig of 4,561,905 bp. The whole genome map of *E. asburiae* L1 generated from the single DNA molecule with the automated Argus system (OpGen Inc.) was aligned with the sequence obtained from the Pacific Biosciences RS II sequencing technology to investigate the mismatch tolerance. Figure 2 showed that although two different sequencing technologies were applied, both sequences generated are highly aligned with each other, confirming the completeness of this genome. Apart from that, the complete genome is proved to be circular with the help of DNAPlotter (version 1.4) and Gepard (version 1.3) (Figure 3).

### 3.3. Gene Prediction and Annotation of E. asburiae L1

**Figure 4.**
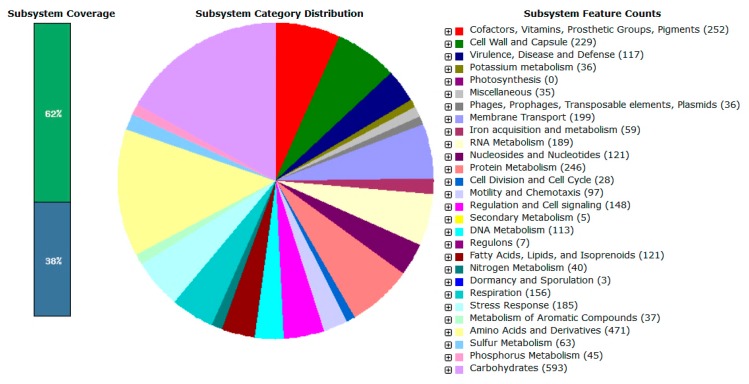
Subsystem category distribution statistics for *E. asburiae* L1. The complete genome sequence of *E. asburiae* L1 was annotated using the Rapid Annotation System Technology (RAST) server. The pie chart showed the count of each subsystem feature and the subsystem coverage. The green bar of the subsystem coverage corresponds to the percentage of the proteins included in the subsystems while the blue bar corresponds to the percentage of the proteins that are not included in the subsystems.

Gene prediction by Prodigal showed that the complete genome of L1 carried 4223 coding DNA sequences (CDS). The data was then annotated using RAST and the subsystem category distribution was shown in Figure 4.

Like most *Proteobacteria*, the majority of L1 genes (593 counts) are responsible for carbohydrate metabolism, followed by amino acids and derivatives; cofactors, vitamins, prosthetic groups and pigment production with 471 and 252 counts, respectively. Generally, these genes are responsible for the basic life-sustaining needs of the bacterial cell. Apart from the presence of the basic necessary genes in L1, there are 117 genes responsible for virulence, disease and defense. Among these 117 genes, 85 were found to play a role in controlling the resistance against antibiotics and toxic compounds.

Previous studies have indicated that members of the *Enterobacteriaceae* cause gastrointestinal illnesses, such as diarrhea. Outbreaks have been reported all around the world, commonly connected to vegetable and fruit products [21,22]. Studies also showed that *Enterobacteriaceae* colonization is believed to trigger spoilage activities in food products [23,24]. Due to the raise of awareness concerning human health, multidisciplinary interest research in the involvement of QS in both food spoilage and food-borne illnesses caused by enteric bacteria has increased. Our data revealed the presence of virulence-related gene *virK*, which has been reported to be an important virulence determinant in other species, especially at the late stages of infections [25,26]. This leads to the speculation that *E. asburiae* L1 might be a pathogen. However, more studies need to be carried out to explore the mechanisms involved in pathogenesis of this organism.

**Figure 5.**
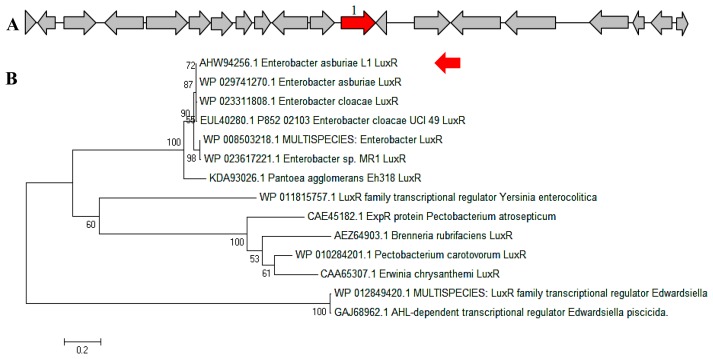
Putative *luxR* gene of *E. asburiae* L1. (**A**) The red arrow showed the visual region of *E. asburiae* L1 *luxR* gene and (**B**) Phylogenetic analysis of *E. asburiae* L1 *luxR* gene. The tree was constructed based on the LuxR protein sequences by Neighbor-Joining with bootstraps value of 1000 replicates.

**Figure 6.**
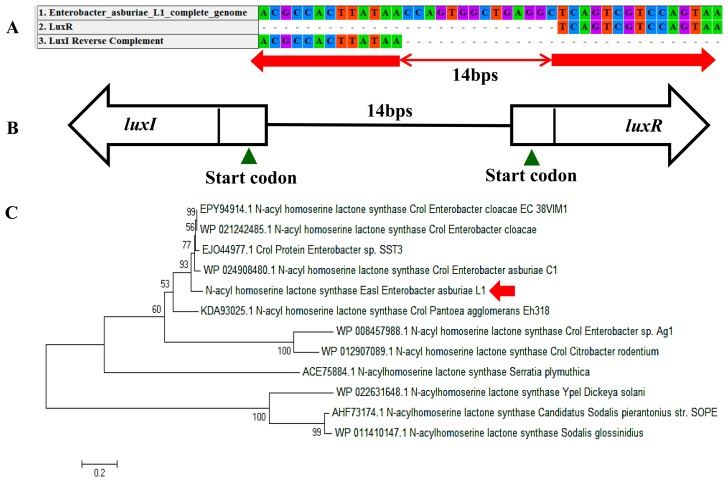
*N*-acyl homoserine lactone synthase EasI and transcriptional regulator of *E. asburiae* L1. (**A**) The alignment of *luxI* and *luxR* genes with 14 bps apart; (**B**) The arrows showed the transcriptional direction of *luxI* and *luxR* genes while the green triangle indicated the site for start codons and (**C**) Phylogenetic analysis of *E. asburiae* L1 *luxI* gene. The tree was constructed based on the similar LuxI protein sequences by Neighbor-Joining with bootstraps value of 1000 replicates.

In a complete AHL-based QS system, the *luxI/luxR* homologs interact with each other whereby AHLs synthesized by LuxI bind to and activate the LuxR-type protein [27]. This AHL-protein complex in turn regulates the expression of certain genes, leading to the collective behaviors of the bacteria [28]. The *luxI/luxR* pairs are often genetically linked. However, there are examples where the *luxI/luxR* functional pairs are distantly located in the bacterial chromosome or plasmid. For instance, *Pseudomonas aeruginosa* has been reported to carry an unpaired *luxR* [27,29] which is responsible for the cognate signaling molecules produced by both its existing AHL synthase and the signaling molecules from the environment [30]. A putative *luxR* gene (Figure 5A) with the size of 693 bps was identified at the location in between 1,633,036 and 1,633,728 of the *E. asburiae* L1 complete genome. In Figure 5B, the phylogenetic analysis based on amino acid sequences showed that the gene *luxR* of *E. asburiae* L1 grouped under the same AHL-based QS transcriptional regulator family as compared with other *E. asburiae* and closely related enterobacteria. Furthermore, an AHL synthase gene of *E. asburiae* L1 with a size of 639 bps was found located in the region in between 1,633,743 and 1,634,381 of this genome. The alignment (Figure 6A) of *luxI* and *luxR* genes sequences showed that they are 14 bps apart with opposite orientation (Figure 6B). To date, reports on the presence of LuxI in *Enterobacter* are still very limited.

Our initial analysis of the *luxI* homolog of *E. asburiae* L1 was annotated as ‘*croI*’ found in *Citrobacter rodentium*. In Figure 6C, the phylogenetic analysis based on amino acid sequences showed that the AHL synthase found in *E. asburiae* L1 formed a separate cluster as compared with other *E. asburiae* and closely related enterobacteria. Therefore, we decided to name the *luxI* homolog of *E. asburiae* L1 *easI*.

According to Rezzonico *et al.*, *E. asburiae* possesses an autoinducer-2 (AI-2)-based QS system. In our present work, genes that have been known to be involved in the AI-2-based system were detected as well. Although the AI-2 QS system has been proven to be functional in *Vibrionaceae*, doubts regarding AI-2 status as a universal signal still remain unresolved to date. In fact, limited studies were conducted to explore the phenotypes regulated by AI-2 in others bacteria apart from *Vibrio harveyi*. According to Winzer *et al.*, molecules such as AI-2 that function as cell-to-cell signals in some organisms may not necessarily do so in others. In addition, the question marks on whether the synthesis of the AI-2 molecules is really catalyzed by an enzyme solely dedicated to its production and whether their primary function as a true QS system in all bacteria are yet to be solved [31,32]. This especially applies for those that occur in bacteria without a *luxS* gene.

On the other hand, AHL-regulated phenotypes have successfully been identified after the inactivation of *luxI* homologue genes [33]. Even though very little is known about the relationship between QS in *E. asburiae* and its pathogenicity, studies have shown that *Enterobacteriaceae* that possess diverse AHL signals are more capable of causing infections [34]. Therefore, compared to AI-2, our interest is more towards study on the AHL-based QS system in *E. asburiae* that has been described in our previous study.

To date, apart from *E. asburiae* L1, only two different strains of *E. asburiae* have been found deposited in DDBJ/EMBL/GenBank, namely *E. asburiae* LF7a (accession CP003026) and *E. asburiae* C1 (accession JACW00000000). Although the genome of *E. asburiae* LF7a has been completely sequenced, no AHL-based regulated QS system was detected in the sequence. In contrast, *E. asburiae* C1, which possessed an AHL-based QS system, is not a complete genome sequence. Consequently, the presence of both AHL synthase *easI* gene and the success in obtaining the complete genome of *E. asburiae* L1 is a great stepping stone for us to move towards exploration of the interaction of the AHLs produced by *E. asburiae* L1 with the virulence genes in order to gain a better understanding on how these interactions may affect food safety and human health.

In addition, there is much recent interest in exploring QS as a novel anti-infectious therapy [35,36] as it does not involve the use of antibiotics. Theoretically, this will reduce drug resistance problems [35]. In fact, a previous study by Dong *et al.* has proved that expression of aiiA in transformed Erwinia carotovora strain SCG1 significantly reduced the amount of autoinducer produced, thus decreasing extracellular pectolytic enzyme activities, and attenuating pathogenicity on potato, Chinese cabbage, celery, carrot, cauliflower, eggplant, and tobacco [37]. In addition, Rasmussen *et al.* showed that blockage of the QS systems attenuates *Pseudomonas aeruginosa* [38]. Therefore, the complete genome of our *E*. *asburiae* L1 isolate will allow us to further investigate the QS-mediated gene expression in this bacterium, as well as the development of novel anti-QS molecules [39–43].

## 4. Conclusions/Outlook

The AHL synthase gene *easI* of *E. asburiae* L1 was discovered in this work thanks to the availability of its complete genome. Analysis of this complete genome also indicated the presence of virulence factor coding genes. It is believed that the virulence factors might be coordinated by QS, so this complete genome may provide insights into the QS-mediated pathogenesis and virulence determinants of this potential pathogen. Therefore, our future work will focus on the AHL-based QS gene regulation of *E. asburiae* L1 to determine the importance and functions of these signaling molecules in the hope of enhancing produce safety and elucidating the mechanisms of QS-regulation in *E. asburiae*.

## 5. Availability of Supporting Data

The complete genome sequence of *E. asburiae* strain L1 was deposited in DDBJ/EMBL/GenBank under the accessions CP007546. The version described in this paper is the first version.
